# Development of an efficient and heritable virus-induced genome editing system in *Solanum lycopersicum*

**DOI:** 10.1093/hr/uhae364

**Published:** 2024-12-28

**Authors:** HuiJun Lee, Ji Eun Baik, Kyung-Nam Kim

**Affiliations:** Department of Bioresources Engineering, Sejong University, Neungdong-ro 209, Gwangjin-gu, Seoul 05006, Republic of Korea; Department of Bioresources Engineering, Sejong University, Neungdong-ro 209, Gwangjin-gu, Seoul 05006, Republic of Korea; Department of Bioresources Engineering, Sejong University, Neungdong-ro 209, Gwangjin-gu, Seoul 05006, Republic of Korea

## Abstract

The CRISPR-Cas9 system can be used to introduce site-specific mutations into the genome of tomato (*Solanum lycopersicum*) plants. However, the direct application of this revolutionary technology to desirable tomato cultivars has been hindered by the challenges of generating transgenic plants. To address this issue, we developed an efficient and heritable genome editing system using tobacco rattle virus (TRV) for an elite tomato cultivar (the paternal line of Saladette). Notably, this virus-induced genome editing (VIGE) system enables the rapid production of various mutant seeds without the need for additional plant transformation and tissue culture, once a Cas9-expressing tomato line is established. This VIGE system consists of transgenic tomato plants that express Cas9 under the control of the tomato ubiquitin 10 (*SlUbi1*0) gene promoter and a mobile guide RNA scaffold (gRNA:SlmFT) generated using the sequence of the tomato *Flowering Locus T* (*SlFT*) gene. We determined its editing efficiency by targeting the tomato phytoene desaturase (*SlPDS*) gene, which causes photobleaching symptoms when disrupted. Most transgenic seedlings infected with the TRV vectors carrying the *SlPDS-*targeting sgRNA developed chimeric albino leaves associated with a high frequency of indel mutations in the *SlPDS* gene. Remarkably, fruits from these plants yielded homozygous *SlPDS* knockout seeds at rates ranging from 15% to 100%. These results demonstrate the exceptional effectiveness of our VIGE system in rapidly generating heritable genome edits in tomato.

## Introduction

The clustered regularly interspaced short palindromic repeats (CRISPR)/CRISPR-associated protein (Cas) machinery is an adaptive immune system in prokaryotes for the defense against invading viruses and plasmids [1]. Engineering of this system has enabled targeted genome editing in a variety of eukaryotes [2]. It requires the expression of two key components in the cells: Cas endonucleases and a single guide RNA (sgRNA) carrying a target sequence of 20 nucleotides. The sgRNA directs the Cas enzyme like Cas9 and Cas12 to cut the target chromosomal DNA at a specific site complementary to the 20-nucleotide sequence followed by the protospacer-adjacent motif (NGG). The site of double-strand DNA break in the genome can be repaired by a non-homologous end-joining mechanism, which is error-prone and often causes mutations (either insertions or deletions; indels) during the process [3, 4]. The simplicity and high efficiency of the CRISPR-Cas system facilitates genome manipulation with unparalleled ease compared to the previous genome editing technologies such as zinc finger nucleases [5] and transcription activator-like effector nucleases [6].

Plant-specific CRISPR-Cas9 vectors have been developed in a few initial studies and applied to several model species such as *Arabidopsis thaliana*, *Nicotiana benthamiana*, and *Oryza sativa* to successfully generate desired mutants with heritable genomic modifications [7–9]. Since then, this innovative and sophisticated genome-editing technology has been widely adopted to create a variety of targeted mutations in numerous crops, including rice, maize, wheat, cotton, lettuce, potatoes, soybeans, and tomatoes [10–12]. However, it is important to note that in these studies, genome-edited crops with intended mutations were mostly achieved using conventional tissue culture techniques, which not only require lengthy regeneration periods but also work only for a limited range of plant species and varieties. Consequently, these constraints have hindered the application of CRISPR-Cas9 system to plant species that are recalcitrant to stable transformation.

**Figure 1:**
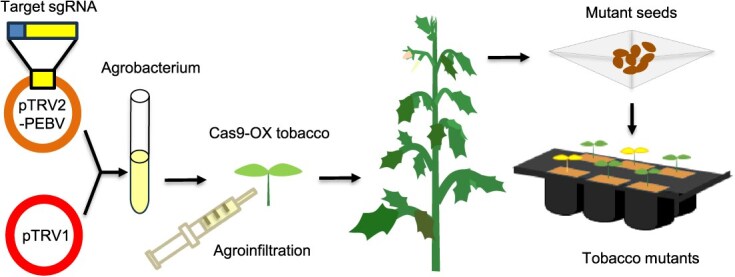
Overview of the tobacco rattle virus-mediated genome editing system in tobacco plants. A 20-nucleotide target sequence is cloned before the sgRNA scaffold under the PEBV promoter in the pTRV2-PEBV plasmid. Agrobacterium (GV3101) cultures harboring both pTRV1 and the engineered pTRV2-PEBV vectors are co-infiltrated into the cotyledons of Cas9-expressing tobacco plants. After agro-infiltration, their systemic leaves are analyzed for the presence of indel mutations at the targeted site. Eventually, tobacco mutants are isolated by screening the harvested seeds, eliminating the need for additional tissue culture

To address these issues, several viruses, such as potato virus X (PVX), sonchus yellow net rhabdovirus, and tomato spotted wilt virus (TSWV), have been engineered to deliver Cas9 and sgRNAs into plant [13–15]. In particular, a tobacco rattle virus (TRV)-mediated genome editing system has been developed to reduce the reliance on tissue culture procedures for producing genome-edited plants with targeted modifications [16, 17]. Basically, this virus-induced genome editing (VIGE) system consists of Cas9-expressing tobacco (*N. benthamiana*) transgenic lines and TRV-derived sgRNA delivery vectors (Fig. 1).

Since TRV possesses a bipartite RNA genome (RNA1 and RNA2), two TRV-based vectors were constructed: pTRV1 vector primarily contains RNA-dependent RNA polymerase and movement protein genes under the control of two copies of 35S promoter sequences in a T-DNA binary vector, pCAMBIA0390. The RNA2 genome is cloned into another type of T-DNA binary vector, pBIN19, creating the pTRV2-PEBV plasmid. This plasmid harbors the pea early browning virus (PEBV) promoter, which drives the expression of the guide RNA (gRNA) scaffold [17]. This VIGE system begins with cloning a user-determined target sequence in front of the gRNA region to make a target-specific sgRNA in the pTRV2-PEBV vector. Then, pTRV1 and sgRNA-carrying pTRV2-PEBV plasmids are co-introduced into the transgenic tobacco leaves via Agrobacterium infiltration to reconstitute the TRV virus, allowing the expression of the sgRNA that can direct Cas9 nuclease to cleave the target DNA sequence in the tobacco genome. Since TRV had been reported to have the ability to invade the shoot apical meristem [18], the infected tobacco plants were expected to produce seeds with heritable mutations in the target site. Contrary to this expectation, however, the VIGE system yielded mutant seeds at a very low frequency; only one mutant was identified among 438 harvested seeds [17].

The low heritable editing efficiency of the VIGE system was dramatically improved in tobacco by fusing the *Flowering Locus T (FT)* gene of *A. thaliana* to the 3′-end of the sgRNA scaffold [19]. This modification resulted in a dramatic increase in mutant seed production, with rates ranging from 65% to 100% in the following generation. Arabidopsis *FT* mRNA is a mobile RNA that migrates from the leaf vascular tissue to the shoot apical meristem to trigger the onset of flowering [20]. Because mRNAs tagged with the *FT* sequence also become mobile [21], the chimeric sgRNAs are likely to reach the shoot apical meristem more efficiently, leading to a higher frequency of genetic mutations. Unfortunately, however, the application of this VIGE system to tomato was reported to be unsuccessful in generating mutant seeds [22].

As a matter of fact, our recent attempts to directly apply this virus system to a tomato cultivar (Moneymaker) also failed to produce mutant progeny. In this study, therefore, we aimed to develop an efficient and heritable VIGE system specifically designed for tomato plants. To achieve this goal, we first generated transgenic tomato (the paternal line of Saladette cultivar) plants expressing Cas9 in the shoot apical meristem by employing the promoter sequence of the tomato ubiquitin 10 gene (*SlUbi1*0). Next, we identified and utilized the tomato *FT* gene (*SlFT*) to construct a mobile sgRNA. We then verified the effectiveness of this modified system by targeting the phytoene desaturase gene in tomato (*SlPDS*). Fortunately, these approaches successfully yielded a substantial number of *SlPDS* knockout mutant seeds without requiring tissue culture, underscoring the exceptional efficacy of our genome-editing system in introducing precise genetic modifications into the tomato genome. Consequently, this tomato-optimized VIGE platform is a highly versatile and efficient tool capable of rapidly generating diverse tomato mutants.

## Results

### Identification and cloning of the *SlUBI10* promoter

The TRV-based VIGE system has been shown to efficiently produce specific genetic mutations in tobacco (*N. benthamiana*) at a high frequency [23]. However, when we applied this genome-editing system directly to tomato transgenic plants that express Cas9 under the control of Arabidopsis UBI10 promoter, no mutant seeds were produced (data not shown). We considered two possible explanations for this failure. First, the Arabidopsis UBI10 promoter may not be suitable for driving Cas9 expression in the shoot apical meristem and germline cells of tomato. Second, the Arabidopsis *FT* mobile sequence may not be effective in transporting sgRNA from the cotyledons to the shoot apical meristem of tomato. To achieve efficient and heritable genome editing with the VIGE system, it is crucial for both sgRNA and Cas9 to be present simultaneously in the shoot apical meristems.

**Figure 2:**
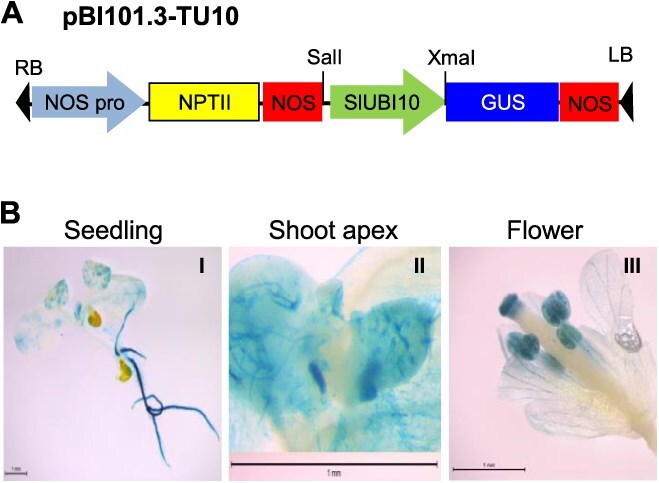
Cloning of the 5′ upstream sequence of the tomato Ubiquitin10 (SlUBI10) gene and its promotor activity in Arabidopsis. (A) Schematic diagram of the *SlUBI10* promoter::GUS construct in pBI101.3 plasmid, pBI101.3-TU10. RB and LB refer to the right and left borders of the T-DNA, respectively. (B) Histochemical GUS staining of the transgenic Arabidopsis plants carrying the pBI101.3-TU10 plasmid. I, 2-week-old seedling; II, shoot apex; III, flower. Scale bars, 1 mm. Six independent transgenic plants were examined, and five of which exhibited similar patterns of GUS expression

To address this issue, we first attempted to search for a strong tomato promoter that can drive Cas9 expression in the shoot apical meristem and germline cells. Such promoter characteristics are considered critical for efficiently generating heritable mutations in plants via the VIGE system. To this end, we isolated a 2148-bp DNA fragment upstream of the tomato ubiquitin10 gene (*SlUBI10*; Solyc10g006480) and investigated its promoter activity. We fused the 5′ flanking sequence to the β-glucuronidase (GUS) reporter gene in the pBI101.3 binary vector, generating the construct pBI101.3-TU10 (Fig. 2A). Histochemical analysis (Fig. 2B) of Arabidopsis transgenic plants harboring this chimeric plasmid revealed strong GUS expression throughout the shoots and roots. Furthermore, it is noteworthy that the *SlUBI10* promoter region showed relatively high activity in the shoot apical meristems and stamens, which is crucial for the successful implementation of the VIGE system.

**Figure 3:**
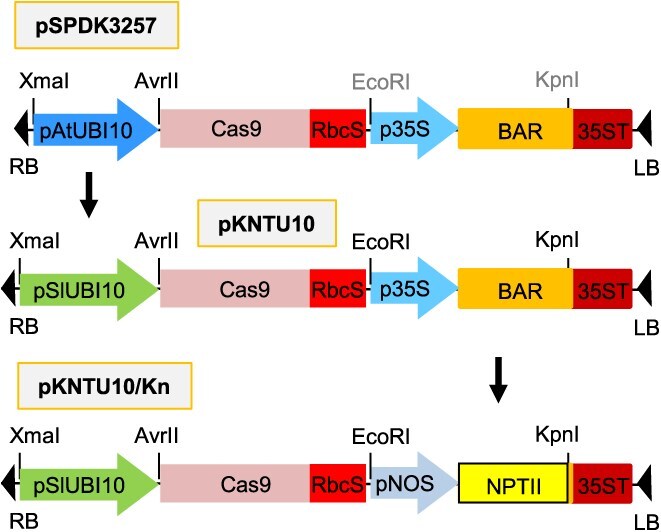
Schematic diagrams of the pKNTU10/Kn plasmid construction. The pSPDK3257 plasmid, kindly provided by Prof. Dinesh-Kumar at UC Davis, is an *Agrobacterium* binary vector: RB and LB represent the right border and left border of the T-DNA, respectively. AtUBI10, promoter of the Arabidopsis ubiquitin 10 gene; Cas9, CRISPR-associated protein 9; RbcS, terminator of the ribulose-1,5-bisphospate carboxylase small subunit gene; p35S, CaMV 35S promoter; BAR, Basta (glufosinate ammonium)-resistance gene; 35ST, CaMV 35S terminator; pSlUBI10, promoter of tomato ubiquitin 10 gene (LOC101248251; Solyc07g064130); pNOS, promoter of nopaline synthase gene; NPTII, neomycin phosphotransferase II promoter

Therefore, we replaced the Arabidopsis *UBI10* promoter in the pSPDK3257 vector with the tomato *SlUBI10* promoter, resulting in the construction of the pKNTU10 plasmid (Fig. 3). This substitution is expected to induce Cas9 expression in the shoot apical meristem and germline cells, thereby enhancing the efficiency of heritable genome editing in tomato using the VIGE system. In addition, the pKNTU10 vector was further modified to the pKNTU10/Kn plasmid by replacing the p35S::BAR region with pNOS::NPTII fusion gene. This modification was made because our previous experience indicated that kanamycin is a more effective selection agent than the herbicide glufosinate for tomato transformation and regeneration.

### Generation of transgenic tomato plants that express Cas9

**Figure 4:**
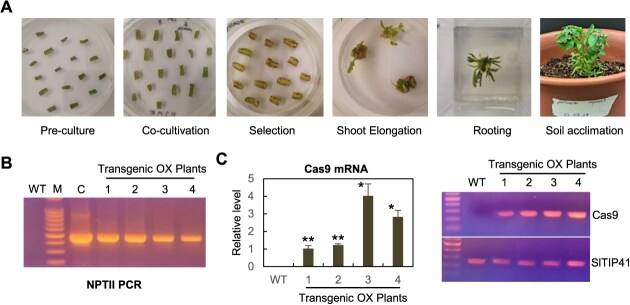
Development of transgenic tomato (the paternal line of Saladette) plants that express Cas9 gene. (A) Photos showing each step of the tomato transformation process mediated by Agrobacterium strain (LBA4404) carrying the pKNTU10/Kn plasmid. (B) PCR analysis verifying T-DNA integration in the nuclear genome of transgenic tomato plants. Genomic DNAs extracted from the indicated plants were PCR-amplified with a set of NPTII primers, NPTII-F and NPTII-R. WT, wild-type tomato; M, DNA size marker; C, pKNTU10/Kn plasmid control; OX 1 ~ 4, independent transgenic tomato lines. (C) Real time RT-qPCR assays showing the transcript levels of Cas9 gene in the transgenic plants (left panel). For Cas9 gene, Cas9-RT-F2 and Cas9-RT-R2 primers were designed to produce 214-bp PCR product. The tomato housekeeping gene *SlTIP41* (Solyc10g049850) was co-amplified to yield a 235-bp PCR product using SlTIP41-RT-F and SlTIP41-RT-R primers, which served as an internal control. The Cas9 expression level in the transgenic line 1 was set to 1.0 to display relative abundance difference. Error bars denote standard deviation (SD) of three biological replicates. Significance was calculated using the Student's *t*-test: ^*^P < 0.05, ^**^P < 0.01. Agarose gels (right panel) show the products of the RT-qPCR

We generated transgenic tomato plants (the paternal line of Saladette) that express the Cas9 gene under the control of the *SlUBI0* promoter, using the *Agrobacterium* strain LBA4404 harboring the pKNTU10/Kn plasmid. An example of the *Agrobacterium*-mediated plant transformation process we performed is illustrated in Fig. 4A. The transgenic tomato plants, initially isolated with kanamycin selection, were further confirmed by PCR analysis to demonstrate the integration of the NPTII gene in their genome (Fig. 4B). Four independent T0 transformants were eventually selected via these steps and self-pollinated for two generations to obtain homozygous transgenic lines.

To determine the Cas9 expression levels, we subsequently performed real-time reverse transcription quantitative PCR (RT-qPCR) analysis on the transgenic tomato plants. As expected, all transgenic lines exhibited significant expression of the Cas9 gene, although there were some variations in expression levels among them (Fig. 4C). Since transgenic line #3 displayed a higher level of Cas9 expression than the others, it was selected for the subsequent VIGE experiments.

### Construction of mobile sgRNA using tomato *SFT* mRNA

**Figure 5:**
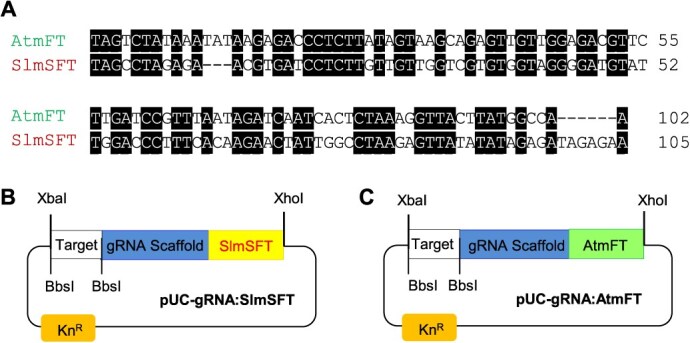
Construction of mobile sgRNA entry vectors using Arabidopsis *FT* or tomato *SFT* genes. (A) Comparative sequence analysis of the mobile elements identified in the FT genes of Arabidopsis and tomato, designated AtmFT and SlmSFT, respectively. To eliminate the possibility of translation, the first two bases were changed from AT to TA. Conserved nucleotides are highlighted with a black background, and gaps are introduced as dashes to optimize alignment. DNA sequences were analyzed with the MegAlign software from DNASTAR. (B) Schematic display of the pUC-gRNA:SlmSFT entry plasmid. The tomato *FT* (SlmSFT; Solyc03g063100.2.1) mobile sequence was included at the 3′ end of the gRNA scaffold. (C) Schematic representation of the pUC-gRNA:AtmFT entry plasmid. The mobile sequence of Arabidopsis *FT* gene (AtmFT, AT1G65480) was added to the 3′ end of gRNA scaffold in the pUC-GW-Kan plasmid. Kn^R^ indicates the kanamycin resistance gene

Next, we addressed the inefficiency of the Arabidopsis *FT* mobile sequence in tomato. It has been demonstrated that the Arabidopsis *FT* sequence can dramatically increase the frequency of heritable mutations in tobacco when fused to sgRNAs. [19]. However, as mentioned before, our previous attempts to improve CRISPR-Cas9 editing efficiency in tomato using the Arabidopsis mobile element were unsuccessful. These results were consistent with a recent report [22]. Together, these findings strongly suggested that the Arabidopsis *FT* sequence might be ineffective in transporting sgRNAs to the shoot apical meristem of tomato plants. Therefore, in this study, we attempted to identify a mobile gene in tomato, which can be utilized to develop an efficient sgRNA delivery system. To achieve this, we conducted a BLAST search using the Arabidopsis *FT* gene (AT1G65480) as a query against the tomato genome database on the Solanaceae Genomics Network. This search identified a tomato orthologue, *Single Flower Truss* (*SFT*; Solyc03g063100.2.1) [24], which shares 73.8% overall nucleotide sequence identity with Arabidopsis *FT*. A comparison of the deduced amino acid sequences of the two genes revealed an even higher degree of conservation, with 79% identity and 90% similarity, suggesting a close evolutionary relationship and possibly identical functions. However, it is important to note that the nucleotide sequence of the Arabidopsis *FT* mobile element, which is essential for its mobility [19], shares only 54% identity with the corresponding region in the tomato *SFT* gene (Fig. 5A). The divergence in the mobile sequences of the two *FT* genes may explain why the Arabidopsis *FT* mobile element failed to function properly in tomato.

Based on the sequence analysis, we hypothesized that the mobile element of the tomato *SFT* gene could be used to significantly enhance the mobility of sgRNAs in tomato. To test this, we constructed the mobile sgRNA entry vector pUC-gRNA:SlmSFT by incorporating a 105-bp mobile sequence from the tomato *SFT* gene into the 3′ end of the gRNA scaffold (Fig. 5B). It should be noted that the start codon (ATG) in the tomato *SFT* mobile sequence was changed to TAG to eliminate potential translation in tomato plants. A target nucleotide sequence can be cloned between the BbsI restriction enzyme sites in the entry vector. The sgRNA regions can then be excised using the XbaI and XhoI restriction enzymes and placed under the PEBV promoter in the pTRV2-PEBV destination vector. As a control, we also created the pUC-gRNA:AtmFT construct in a similar manner by fusing the 102-bp Arabidopsis mobile sequence to the 3′ end of the gRNA scaffold (Fig. 5C).

### Development of *SlPDS* knockout mutants using the VIGE platform

**Figure 6:**
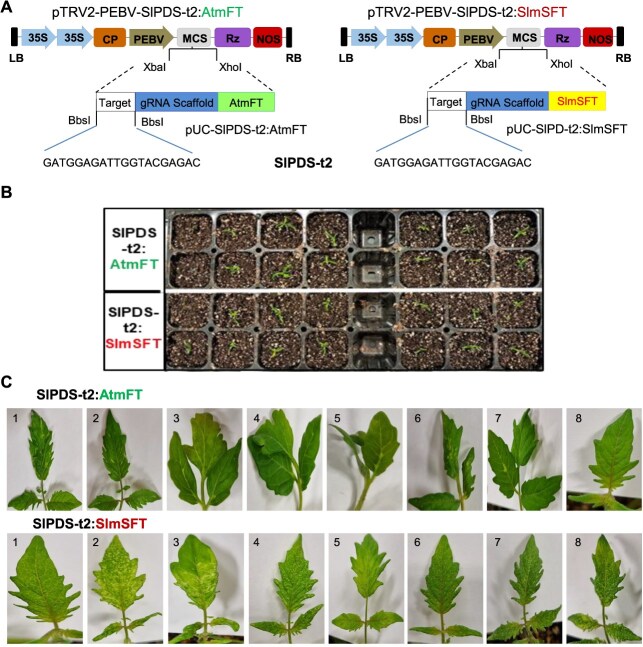
Efficiency test of the established VIGE system for heritable mutation. (A) Schematic diagrams of the pTRV2-PEBV destination plasmids carrying two types of mobile sgRNAs targeting the tomato PDS gene. LB and RB indicate left and right borders of T-DNA. 35S, CaMV 35S promoter; CP, coat protein; PEBV, pea early browning virus promoter; MCS, multiple cloning sites; Rz, self-cleaving ribozyme; NOS, nopaline synthase terminator. AtmFT and SlmSFT represent Arabidopsis and tomato mobile FT sequences, respectively. SlPDS-t2 indicates SlPDS-targeting nucleotide sequence. (B) Photos taken 4 days after agroinfiltration. The cotyledons of Cas9-expressing tomato seedlings were infiltrated with Agrobacterium strain (GV3101) carrying the indicated mobile sgRNAs. (C) Systemic leaves were observed 3 weeks after agroinfiltration. White spots suggest loss of SlPDS function. Eight plants were selected and assigned the numbers for further analysis. Similar results were observed from all three independent biological replicates

To investigate the heritable genome-editing efficiency of the VIGS platform established above, we constructed two plant destination vectors, pTRV2-PEBV-SlPDS-t2:AtmFT and pTRV2-PEBV-SlPDS-t2:SlmSFT, designed to target the tomato phytoene desaturase gene (*SlPDS*). As shown in Fig. 6A, both vectors share the same 20-bp target recognition sequence (SlPDS-t2) but differ only in the mobile elements attached to the 3′ end of the sgRNA; AtmFT and SlmSFT. The *SlPDS* gene was chosen as a target due to its visually distinct photobleaching phenotype upon mutation. Disruption of *PDS* function results in this phenotype primarily because it impairs the production of photoprotective carotenoids [25].

Using the agro-infiltration method, we separately introduced the two pTRV2-PEBV-SlPDS-t2 vectors (tagged with either the AtmFT or SlmSFT mobile element), along with the helper plasmid pTRV1, into the cotyledons of Cas9-expressing tomato seedlings derived from the transgenic line OX-3 (Fig. 6B). Approximately, three weeks after infiltration, the tomato seedlings infected with the SlPDS-t2-SlmSFT sgRNA developed new leaves with photobleached spots, whereas no photobleaching phenotype was observed in the control seedlings treated with the SlPDS-t2-AtmFT sgRNA (Fig. 6C). The results suggested that, unlike its Arabidopsis counterpart, the tomato *SFT* mobile element appears to be effective in making sgRNA available at the shoot apical meristem, thereby increasing the genome editing efficiency in the systemic leaves of tomato. It is quite intriguing that the Arabidopsis *FT* mobile element, which was effective in tobacco (*N. benthamiana*) [23], failed to exhibit a similar effect in tomato, a member of the Solanaceae family like tobacco. Nevertheless, this finding is consistent with a recent study that reported the ineffectiveness of the Arabidopsis *FT* sequence in inducing mutations in the systemic leaves of tomato [22].

Targeted deep sequencing analysis was performed on the plants shown in Fig. 6C and the wild-type control to evaluate the efficiency of *SlPDS* gene editing in systemic leaves. Genomic DNA was extracted from two systemic leaves of each plant and used as a template for PCR-amplification of the *SlPDS* target region. The PCR products were then analyzed by next-generation sequencing (NGS). NGS analysis indicated that the indel mutation rates of the *SlPDS* gene in tomato plants infiltrated with Arabidopsis mFT-tagged sgRNA (SlPDS-t2-AtmFT) did not significantly differ from those of the wild-type control. In contrast, tomato plants treated with sgRNA attached with tomato mSFT (SlPDS-t2-SlmSFT) exhibited dramatically increased indel frequencies, ranging from 20% to 71% (Table 1A).

**Table 1:** Targeted deep sequencing analysis of the *SlPDS* gene in the tomato plants infiltrated with Agrobacterium carrying *SlPDS-*targeting sgRNA.

**A**						
Sample	Total sequences	With both indicator sequences	More than minimum frequency	Insertions	Deletions	Indel frequency
WT	237 119	14 532	14 177	0	2	2 (0.0%)
SlPDS-t2-AtmFT-1	271 819	25 221	24 739	12	47	59 (0.2%)
SlPDS-t2-AtmFT-2	234 849	32 927	32 306	26	11	37 (0.1%)
SlPDS-t2-AtmFT-3	81 793	36 303	35 659	0	0	0 (0.0%)
SlPDS-t2-AtmFT-4	173 012	36 387	35 685	0	0	0 (0.0%)
SlPDS-t2-AtmFT-5	206 242	32 396	31 789	0	2	2 (0.0%)
SlPDS-t2-AtmFT-6	126 927	20 016	19 610	16	33	49 (0.2%)
SlPDS-t2-AtmFT-7	236 532	31 228	30 623	0	0	0 (0.0%)
SlPDS-t2-AtmFT-8	141 533	37 554	36 895	0	1	1 (0.0%)
SlPDS-t2-SlmSFT-1	256 743	85 735	82 858	9177	27 451	36 628 (44.2%)
SlPDS-t2-SlmSFT-2	219 380	26 857	25 944	8368	10 038	18 406 (70.9%)
SlPDS-t2-SlmSFT-3	186 443	16 405	15 586	3240	4914	8154 (52.3%)
SlPDS-t2-SlmSFT-4	248 131	31 603	30 460	5554	8915	14 469 (47.5%)
SlPDS-t2-SlmSFT-5	319 646	72 788	69 665	11 637	20 017	31 654 (45.4%)
SlPDS-t2-SlmSFT 6	175 517	64 131	63 427	3319	9321	12 640 (19.9%)
SlPDS-t2-SlmSFT 7	114 576	49 825	49 116	5624	11 516	17 140 (34.9%)
SlPDS-t2-SlmSFT 8	237 647	67 594	66 575	10 621	21 360	31 981 (48.0%)
**B**						
$\includegraphics{\bwartpath uhae364fx1}$						

(A) Summary of indel frequencies obtained from NGS analysis of the *SlPDS* gene in the specified tomato samples. (B) Pattern analysis was conducted for indels in the two tomato plants (SlPDS-t2:SlmSFT-2 and SlPDS-t2:SlmSFT-3 lines) exhibiting higher indel frequencies

Pattern analysis of the indels was conducted on the top two lines, #2 (70.9%) and #3 (52.3%), based on their high indel frequencies (Table 1B). It revealed that the predominant mutation in line #2 was a ‘GA’ deletion, followed by a ‘T’ addition. However, the most abundant mutation in line #3 was an ‘A’ addition, with a ‘GA’ deletion occurring less frequently. Notably, both plants displayed a variety of indel mutations. Overall, this NGS analysis confirmed that the photobleaching phenotype was indeed associated with the loss of *SlPDS* function in the tomato plants.

**Figure 7:**
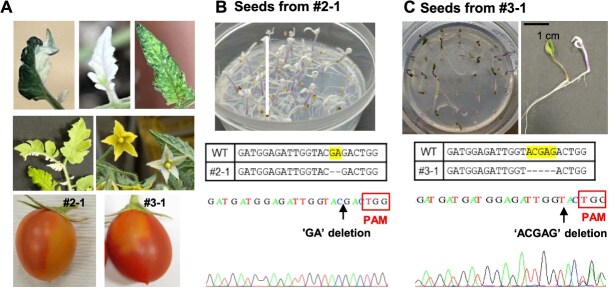
SlPDS knockout mutants were directly obtained via the VIGE platform. (A) Various tissues (leaves, flowers, and fruits) of the SlPDS-t2-SlmSFT-2 (#2) and SlPDS-t2-SlmSFT-3 (#3) tomato plants displayed photobleaching phenotypes. Fruits from the #2 and #3 plants were designated as #2–1 and #3–1, respectively. (B) All the seeds from the #2–1 fruit generated completely bleached seedlings (top panel). Sanger sequencing of the *SlPDS* gene in the white seedlings demonstrated a ‘GA’ deletion (bottom panel). (C) Approximately 50% of the seeds from the #3–1 fruit exhibited a complete *SlPDS* knockout phenotype (top panel). Genomic DNA extracted from an albino seedling was subjected to Sanger sequencing, which revealed an ‘ACGAG’ deletion in the target gene (bottom panel). PAM represents a protospacer adjacent motif (NGG)

We next examined whether the tomato plants #2 and #3, which displayed higher indel frequencies in systemic leaves compared to the others, could produce *SlPDS* knockout mutant seeds. As the plants grew, photobleaching phenotypes appeared in various parts of newly emerged organs, including leaves, flowers, and fruits (Fig. 7A). Remarkably, all the seeds harvested from the partially bleached fruit of the #2 line (#2–1) developed into entirely white seedlings, suggesting a homogeneous knockout of the *SlPDS* gene (Fig. 7B). These albino lines were genotyped by Sanger sequencing and confirmed to have a homozygous ‘GA’ deletion mutation in the targeted region, which was identified the most predominant mutation in the systemic leaves of the tomato #2 line according to the NGS analysis. In the case of the #3–1 bleached fruit harvested from the tomato #3 plant, approximately 50% of the seeds generated fully albino seedlings, which were found to be caused by an ‘ACGAG’ deletion in the *SlPDS* gene (Fig. 7C). Meanwhile, it is noteworthy that other fruits from the #2 and #3 plants, despite exhibiting little to no bleaching phenotype, also contained some seeds that developed into completely white seedlings at a rate of 15% to 25% (Supplemental Fig. S1). Taken together, these results demonstrated that our VIGE system can efficiently and rapidly generate genome-edited seeds in tomato.

## Discussion

Tomato is a globally important crop with significant economic value. It belongs to the *Solanaceae* family, which also includes other economically valuable plants such as tobacco (*N. tabacum*), potato (*Solanum tuberosum*), eggplant (*S. melongena*), and pepper (*Capsicum annuum*). Using Agrobacterium-mediated plant transformation, CRISPR-Cas9 components have been successfully applied to several tomato varieties, enabling rapid and precise improvements in diverse agronomic and horticultural traits [26–28]. However, despite these successes, this traditional gene transfer method is both time-consuming and labor-intensive, and it is not effective in many other commercially valuable tomato varieties, thereby limiting the broader application of this genome-editing system. Therefore, these drawbacks associated with generating stable transgenic tomato plants have prompted the development of alternative approaches for introducing CRISPR-Cas9 components into tomato plant cells to facilitate or improve targeted genome editing.

Plant viruses, such as TSWV and TRV, have been used to develop VIGE systems as an alternative method to overcome the limitations of traditional transgenesis. Liu et al. developed a non-transgenic CRISPR-Cas9 delivery system based on TSWV, an RNA virus with a broad host range exceeding 1000 plant species [15]. The virus was engineered to carry the Cas9 nuclease and sgRNA without compromising its ability to infect plants by removing components essential for insect transmission. Although TSWV-derived vectors efficiently induced somatic gene mutations in tomato, they were unable to produce mutant seeds because TSWV cannot invade germline cells. Therefore, the virus-infected tissues were subjected to *in vitro* culture without antibiotic selection to generate heritable mutations. It has been reported that many dicotyledonous plants exhibit favorable tissue culture responses and can regenerate effectively in the absence of selection pressure [29]. However, the heavy reliance on tissue culture to produce heritable mutants for each target gene can significantly restrict the applicability of TSWV-mediated VIGE for tomato genome editing. This is particularly true for many tomato cultivars and varieties that are recalcitrant to in vitro regeneration. Besides, the high virulence and broad host range of TSWV pose a significant risk of environmental contamination. To reduce this risk, the antiviral agent ribavirin should be included in the tissue culture process to prevent the virus from infecting regenerating cells [15].

TRV-based genome editing systems offer several advantages over TSWV viral vectors. First, unlike TSWV vectors, they do not require antiviral chemicals like ribavirin due to their lower infectivity and milder pathogenicity [22, 30]. Second, TRV does not integrate into the host plant’s genome, thereby eliminating the risk of unintended genetic modifications in subsequent generations [22, 23, 31]. Third, the virus can be easily introduced into *Solanaceae* plants via Agrobacterium. Fourth, TRV can infect germline cells, allowing for the collection of mutant seeds from infected plants [17, 18]. However, the small genome size of TRV presents a limitation. Since TRV cannot accommodate the Cas9 gene, it has been modified into viral vectors that carry only sgRNA molecules. As a result, a transgenic plant expressing Cas9 is necessary for TRV-mediated genome editing in plants [19]. Despite this limitation, the TRV-based VIGE system, combined with the mobile sgRNA, provides a rapid and efficient method of generating diverse mutant seeds with the desired modifications, once a Cas9-expressing plant is established. For example, Cas9-transgenic tobacco (*N. benthamiana*) plants efficiently produced PDS mutant seeds when infiltrated with TRV vectors carrying PDS-targeting sgRNAs tagged with mobile sequences from the Arabidopsis *FT* gene [23]. It is important to note that the ability of the Arabidopsis *FT* sequence to promote heritable mutagenesis in tobacco has greatly enhanced the effectiveness of the TRV-mediated VIGE system, completely eliminating the need for repeated tissue culture cycles to generate genome-edited mutant seeds for each new target.

In the case of tomato plants, however, TRV-mediated VIGE systems have not been successful in producing mutant seeds directly from a Cas9-transgenic line without the tissue culture process. Even though sgRNAs tagged with the Arabidopsis *FT* sequence at the 3′ terminus were used, tomato transformants (cv. Micro-Tom) expressing Cas9 under the control of the maize ubiquitin promoter did not show elevated mutation rates in either somatic or germline cells [22]. The ineffectiveness of the Arabidopsis FT mobile element did not appear to be restricted to the TRV system, as heritable genome editing was not achieved with a PVX vector system in tomato, even with the inclusion of the Arabidopsis FT mobile sequence [32]. Therefore, we hypothesized that the absence of mutant seeds in tomato plants using the TRV-mediated VIGE system might be due to an inadequate promoter for Cas9 expression and/or the inability of the Arabidopsis FT sequence to effectively translocate sgRNA in tomato. It is reasonable to assume that the simultaneous presence of Cas9 and sgRNA in the shoot apical meristem is essential for producing mutant seeds from infected tomato plants without tissue culture when using the VIGE system.

In this study, we addressed these two issues and developed an efficient and heritable TRV-mediated VIGE system tailored for tomato plants. Our initial objective was to isolate a suitable promoter capable of driving high levels of Cas9 expression specifically in the shoot apical meristem of tomato plants. Through extensive analysis, we selected the 5′ upstream region of the *SlUBI10* gene as a promising candidate. Histochemical analysis (Fig. 2) confirmed its strong activity in various tissues, including the shoot apical meristems and stamens. This *SlUBI10* promoter sequence was then employed to drive Cas9 expression in transgenic tomato plants of a commercial tomato variety (the paternal line of Saladette). Transgenic line OX-3, which exhibited the highest level of Cas9 expression, as determined by real-time RT-qPCR analysis (Fig. 4), was ultimately selected for VIGE experiments. Next, we aimed to develop a mobile sgRNA scaffold that would be effective in tomato. We found that the mobile nucleotide sequences of the Arabidopsis *FT* and tomato *SFT* genes are poorly conserved, despite the close evolutionary relationship between the two genes (Fig. 5A). This finding led us to speculate that the Arabidopsis *FT* mobile sequence may not be recognized by the cellular machinery responsible for the movement of the tomato *SFT* mRNA. Therefore, we constructed a mobile sgRNA entry vector (pUC-gRNA:SlmSFT) by incorporating a 105-bp mobile sequence from the tomato *SFT* gene to the 3′ end of the gRNA scaffold, hoping that it would enhance the mobility of the sgRNA in tomato (Fig. 5B).

We verified the effectiveness of these measures by targeting the tomato phytoene desaturase gene (*SlPDS*). Fortunately, our approach proved to be highly effective in generating mutant seeds directly from Cas9-expressing tomato plants, eliminating the need for tissue culture. As shown in Fig. 7, a substantial proportion of seeds harvested from the infected plants germinated into entirely albino seedlings. Overall, our results strongly indicate that the VIGE platform established in this study is a highly efficient and time-saving method for generating a diverse range of genome-edited tomato mutants without undergoing repetitive tissue culture and regeneration processes for each new target gene. Additionally, we would like to highlight the potential for our VIGE system to be applied to a wide range of commercially significant tomato cultivars that have been challenging to regenerate. This can be achieved through introgression with our Cas9-expressing transgenic tomato plants. Therefore, this genome-editing technology, optimized for tomatoes, will significantly expedite the development of novel genotypes with desirable horticultural and agricultural traits.

## Materials and methods

### Plant materials and growth conditions

Seeds of *S. lycopersicum* (cv. the paternal line of Saladette) were supplied by Asia Seed Co., Ltd. (Seoul, Korea). Tomato plants were germinated on MS agar medium containing 3% sucrose at 25°C in a growth chamber under long-day conditions with a 16-h light/8-h dark cycle. After two weeks, the seedlings were transplanted into soil (SunGro Sunshine Mix #4) and cultivated in a greenhouse. *A. thaliana* (ecotype Col-0) plants were grown at 23°C in a growth chamber.

### Genomic DNA isolation and RNA extraction

Genomic DNA was extracted from young plant leaves using a modified cetytrimethylammonium bromide (CTAB) protocol as previously described [33]. Total RNA was extracted from plant tissues using TRIzol reagent (Invitrogen) following the manufacturer’s instruction. The quality and concentration of both DNA and RNA were assessed through spectrophotometric analysis (OD: 260 nm/280 nm).

### Oligonucleotide primers

Primers used in this study for cloning, RT-qPCR, and targeted deep sequencing were listed in Supplemental Table S1. Primers were synthesized by Macrogen Inc. (Seoul, Korea).

### Construction of plasmids

To construct the pBI101.3-TU10 plasmid, a 2148-bp DNA fragment (− 2148 to −1, Supplemental Table S2) upstream of the *SlUBI10* gene (Solyc10g006480) was first PCR-amplified from tomato genomic DNA with SlUbi10-PF1 and SlUbi10-PR1 primers. The PCR product was extracted with a gel extraction kit (Qiagen), and then digested with *Sal*I and *Xma*I restriction enzymes to clone the resulting DNA fragment into the *Sal*I/*Xma*I sites of the pBI101.3 binary vector (Clontech). The pKNTU10/Kn construct was derived from pSPDK3257 (provided by Prof. Dinesh-Kumar, UC Davis) binary vector. First, the Arabidopsis *UBI10* promoter region flanked by *Xma*I and *Avr*II was replaced with the 2148 bp SlUbi10 promoter sequence, which was produced by PCR amplification using the SlUbi10-PF1 (*Xma*I) and SlUbi10-PR1(*Avr*II) primers. Second, the resulting plasmid, designated pKNTU10, was further modified to generate pKNTU10/Kn. This was achieved by replacing the 35S promoter::BAR region, bordered by *EcoR*I and *Kpn*I, with the NOS promoter::NPTII fusion gene. The fusion gene was obtained from the pBI121 plasmid (Clontech) using PCR amplification with NOS-F and KPN-R primers. Both pUC-gRNA:AtmFT and pUC-gRNA:SlmSFT plasmids were custom-made by the COSMO genetech company (Seoul, Korea). The Arabidopsis mFT sequence information was obtained from Ellison et al. [23], and the SlmSFT sequence was derived from tomato *SFT* gene (Solyc03g063100.2.1). DNA sequences of the pKNTU10/Kn and pUC-gRNA:SlmSFT vectors are provided in Supplemental Table S2.

To create the pUC-SlPDS-t2:AtmFT construct, the SlPDS-t2-F and SlPDS-t2-R primers were mixed and allowed to anneal to form double-stranded DNA, which was subsequently digested with *Bbs*I and cloned into pUC-gRNA:AtmFT. Similarly, The pUC-SlPDS-t2:SlmSFT plasmid was constructed by cloning the hybridized primer DNA into the *Bbs*I site of pUC-gRNA:SlmSFT. The pUC-SlPDS-t2:AtmFT and pUC-SlPDS-t2:SlmSFT plasmids were digested with *Xba*I and *Xho*I restriction enzymes, releasing the sgRNA inserts. These inserts were then cloned into the pTRV2-PEBV vector, also known as pSPDK3876 ([23]), yielding pTRV2-PEBV-SlPDS-t2:AtmFT and pTRV2-PEBV-SlPDS-t2:SlmSFT, respectively. All the PCR reactions were performed using Pfu DNA polymerase (Stratagene) to ensure high fidelity. All the constructs mentioned above were validated by Sanger DNA sequencing.

### Generation of transgenic tomato plants

To generate transgenic tomato plants overexpressing Cas9, the pKNTU10/Kn plasmid was transformed into *Agrobacterium tumefaciens* (LBA4404) using the freeze–thaw method [34] and subsequently introduced into tomato plants (the paternal line of Saladette). The transformation procedure was based on a previously described method [35]. The antibiotic-resistant plants were further confirmed for the integration of the NPTII gene into the nuclear chromosome using PCR analysis with NPTII-F and NPTII-R primers.

### Real-time RT-qPCR analysis

Real-time reverse transcription quantitative PCR (RT-qPCR) was conducted using the Rotor-Gene Q system and the QuantiTect SYBR Green RT-PCR kit (Qiagen) as previously described [35]. For Cas9 gene, Cas9-RT-F2 and Cas9-RT-R2 primers were designed to produce 214-bp PCR product. The tomato housekeeping gene *SlTIP41* (Solyc10g049850) was consistently co-amplified to yield 235-bp PCR product using a pair of forward and reverse primers (SlTIP41-RT-F and SlTIP41-RT-R) and served as an internal normalization control [36].

### Selection of *SlPDS-*targeting nucleotide sequence

We initially designed several nucleotide sequences that specifically target the *SlPDS* gene (Solyc03g123760.2) without potential off-targets, utilizing online tools such as CRISPR-P 2.0 [37] and CRISPR RGEN Tools (http://www.rgenome.net). Using the UNAFold web server (RNA Folding Form V2.3; http://www.unafold.org/mfold/applications/rna-folding-form-v2.php), the candidate target sequences were further analyzed for their secondary structure formation to select the most efficient sequence. Following these steps, we eventually identified an optimal target sequence (5′-GATGGAGATTGGTACGAGAC-3′) and designed forward (SlPDS-t2-F) and reverse (SlPDS-t2-R) primers accordingly for sgRNA construction.

### Analysis of *SlUBI10* promoter-GUS expression

The *SlUBI 10* promoter-GUS construct (pBI101.3-TU10) was initially introduced into *A. tumefaciens* (GV3101) and subsequently transformed into *A. thaliana* (Col-0) by the floral dipping method [38]. Transgenic plants were selected on 1/2 MS agar plates containing 50 μg/mL of kanamycin as described previously [39]. For histochemical GUS assays, T1 seedlings were stained with X-gluc (5-bromo-4-chloro-3-indolyl-beta-glucuronide) and washed with 80% ethanol as described [40].

### Agro-infiltration of tomato cotyledons

Using *A. tumefaciens* (GV3101), pTRV1 and pTRV2-PEBV vectors were delivered into Cas9-expressing tomato. The tomato plants should be around 7 to 8 days old after germination, before the true leaves appear. Agro-infiltration was performed as described [41].

### Analysis of somatic cell-editing efficiency by targeted deep sequencing

Genomic DNA was prepared from the third and fourth systemic leaves of agro-infiltrated plants approximately three weeks after infiltration to assess somatic cell editing efficiency. Two 8-mm leaf punches collected from the systemic leaves were combined to extract genomic DNA. This genomic DNA was used as a template to PCR-amplify the SlPDS target site with the primer set, SlPDS-t2-1st-F and SlPDS-t2-1st-R, yielding a 534 bp amplicon. The PCR product was diluted 1:10 and then subjected to a second round of PCR using the nested primers, SlPDS-t2-2nd-F and SlPDS-t2-2nd-R, which carry the Illumina adapter sequences. For NGS, this second-round PCR product was diluted 1:10 and amplified with Illumina index primers (forward: D501 ~ D505, reverse: D701 ~ D705). Each sample was amplified with a different combination of the index primers. These final PCR products were purified using the QIAquick PCR Purification Kit (Qiagen) and sequenced on an Illumina Miseq platform at the KAIST Bio Core Center (Daejeon, Korea). Paired-end fastq files resulting from NGS were analyzed using Cas-Analyzer of CRISPR RGEN Tools (http://www.rgenome.net/cas-analyzer) with a 40-bp comparison range to determine indel frequency as previously described [42].

## Distribution of Materials

Upon request, all novel materials described in this publication will be made available in a timely manner for non-commercial research purposes.

## Supplementary Material

Web_Material_uhae364

## Data Availability

The data underlying this article are available in the article and in its online supplementary material.
