# Supplementary material for: Development of an efficient and heritable virus-induced genome editing system in Solanum lycopersicum
Source: Hortic Res. 2024 Dec 28;12(4):uhae364. doi: 10.1093/hr/uhae364 (PMC11891477; doi:10.1093/hr/uhae364)
Supplement: Web_Material_uhae364 [file web_material_uhae364.zip › Supplemental Figure S1.pdf]

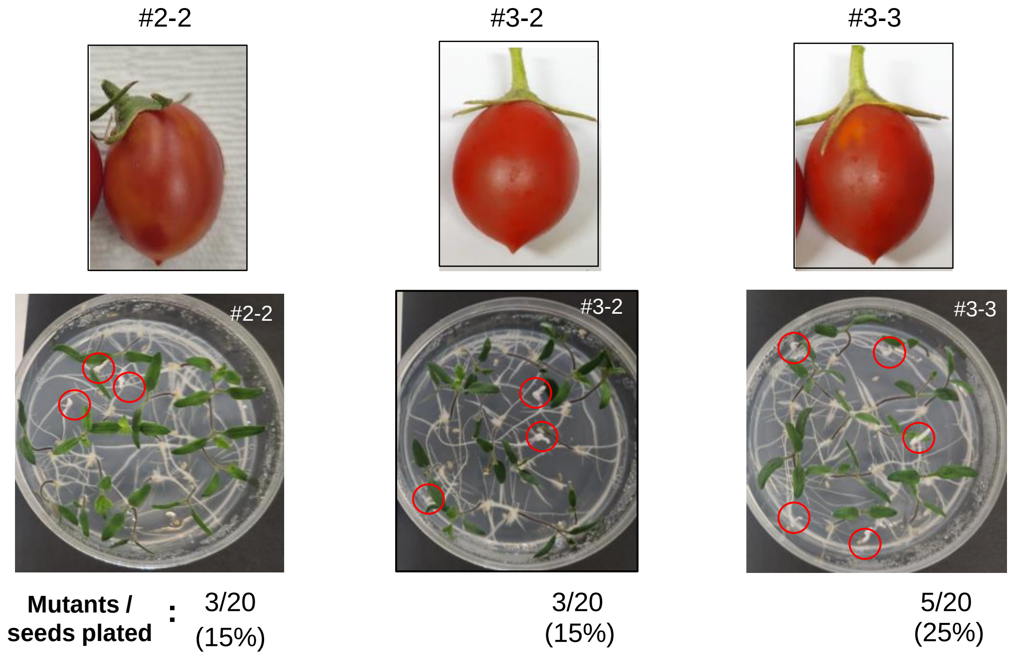

Suppemental Figure S1. Determination of the proportion of the *SlPDS* knockout mutants present in the seeds of the non-bleached fruits. Fruits exhibiting no obvious photobleached phenotype were harvested from the SlPDS-t2-SlmSFT-2 (#2) and SlPDS-t2-SlmSFT-3 (#3) tomato plants and were designated as #2-2, #3-2, and #3-3, respectively (top panels). Twenty seeds derived from the indicated fruits were respectively plated on MS medium supplemented with 50 μg/mL kanamycin and incubated in a growth chamber at 25°C. Photos were taken ten days after germination (bottom panels). Red circles indicate completely white seedlings. The ratios of white seedlings (*SlPDS* mutants) to seeds plated were shown below the bottom panels.
